# Free positions in the second stage of labor for pelvic floor protection: a narrative review

**DOI:** 10.3389/fmed.2026.1716862

**Published:** 2026-03-26

**Authors:** Luyi Yan, Yini Mao, Qinjun Lin, Xueyan Xie, Caixi Chen, Jiayi Zhong, Enfu Tao

**Affiliations:** 1 Department of Delivery Room, Wenling Maternal and Child Health Care Hospital, Wenling, Zhejiang, China; 2 Department of Pharmacy, Wenling Maternal and Child Health Care Hospital, Wenling, Zhejiang, China; 3 Department of Neonatology and NICU, Wenling Maternal and Child Health Care Hospital, Wenling, Zhejiang, China

**Keywords:** biomechanics, free positions delivery, intrapartum care, maternal satisfaction, pelvic floor dysfunction, perineal trauma, second stage of labor

## Abstract

**Objective:**

This narrative review synthesizes current evidence on free positions delivery during the second stage of labor, evaluating its impact on pelvic floor protection, key clinical outcomes, and maternal experience, while also exploring underlying biomechanical and molecular mechanisms.

**Methods:**

A comprehensive but non-systematic literature search was conducted in PubMed, Embase, Cochrane Library, and Web of Science from inception to July 2025., using keywords including “free positions delivery,” “upright position,” “second stage of labor,” “pelvic floor dysfunction,” “perineal trauma,” and related terms. Studies were selected based on relevance to maternal position during the second stage and outcomes related to pelvic floor protection, clinical efficiency, and maternal experience. Both observational and interventional studies were included, with priority given to recent systematic reviews, meta-analyses, and high-quality clinical trials. Evidence certainty was narratively assessed using principles of the Grading of Recommendations Assessment, Development, and Evaluation (GRADE) framework.

**Results:**

Free positions are associated with favorable trends in short-term obstetric outcomes, including reduced duration of the second stage and lower rates of perineal trauma. Maternal satisfaction, sense of autonomy, and comfort are consistently improved. For pelvic floor health, the certainty of evidence differs by time horizon: findings suggest a reduction in early postpartum urinary incontinence, but data supporting a long-term protection against pelvic floor dysfunction remain insufficient.

**Conclusion:**

Current evidence supports consideration of free positions during the second stage of labor as a low-intervention strategy that may improve short-term labor outcomes and maternal experience. Successful implementation requires targeted training for healthcare providers and comprehensive patient education. Future research should prioritize longitudinal studies with standardized outcome measures to strengthen the evidence base for long-term pelvic floor protection.

## 1 Introduction

Pelvic floor dysfunction (PFD), encompassing urinary and fecal incontinence and pelvic organ prolapse, is a significant source of long-term morbidity following vaginal birth, adversely affecting the quality of life for a substantial proportion of women (1–3). It is estimated that one in four women will develop some form of PFD in their lifetime, with vaginal birth being a primary risk factor (4). The economic and personal burden of these conditions is profound, driving a modern obstetric focus not just on the safety of birth, but on the long-term health outcomes of mothers (5, 6).

The second stage of labor is a period of exceptional vulnerability for the pelvic floor (7). The sustained pressure exerted by the fetal descending head can lead to overstretching and injury to the muscles (e.g., levator ani avulsion), fascia, and nerves (8). The lithotomy position, still commonplace in many settings, may exacerbate this risk by concentrating mechanical stress on the perineum, limiting pelvic capacity, and potentiating neuromuscular damage (9, 10).

In contrast, free positions—an umbrella term encompassing all non-supine birthing positions, including upright (standing, squatting, kneeling), lateral, and hands-and-knees positions—are proposed to leverage physiological advantages that may facilitate fetal descentand reduce excessive strain on pelvic floor structures (11–13). Consequently, the adoption of free positions has the potential to shorten labor, reduce perineal trauma, and mitigate the risk of long-term PFD.

Despite international guidelines from the World Health Organization advocating for maternal freedom of position during labor (14–16), a significant gap persists between evidence and practice. This narrative review aims to synthesize the current evidence on the benefits of free positions delivery during the second stage of labor. We will critically examine its impacts on pelvic floor function, clinical efficiency, and maternal satisfaction, thereby synthesizing the current evidence to contribute to the discussion on modern intrapartum care practices.

## 2 Search strategy and selection criteria

Given the narrative nature of this review, we conducted a comprehensive but non-systematic literature search. We searched PubMed, Embase, Cochrane Library, and Web of Science from inception to July 2025 using combinations of terms including “free positions delivery,” “upright position,” “maternal position,” “second stage of labour,” “pelvic floor dysfunction,” “perineal trauma,” and related terms. Articles were selected based on their relevance to maternal positioning during the second stage of labor and its impact on pelvic floor outcomes, clinical efficiency, and maternal experience. Both observational and interventional studies were considered, with priority given to recent systematic reviews, meta-analyses, and high-quality clinical trials. Reference lists of relevant reviews were also screened for additional publications. Consistent with the narrative format, we did not apply formal systematic screening procedures or risk-of-bias assessment tools.

To provide readers with a sense of evidence certainty across outcome domains, we performed a narrative assessment informed guided by the principles of the Grading of Recommendations Assessment, Development, and Evaluation (GRADE) framework (17). This was used as a conceptual lens to structure our discussion of evidence strength, rather than as a formal grading exercise.

## 3 International context and development of free positions delivery

### 3.1 Historical evolution and theoretical basis

The World Health Organization’s 1996 Practical Guide for Care in Normal Birth advocated for maternal freedom of movement and position throughout labor to enhance comfort and facilitate spontaneous birth (14). This was reinforced in the 2018 WHO guidelines, which promoted non-supine positions (i.e., any birthing position other than lying on the back, including upright, lateral, and hands-and-knees positions) as a key strategy to reduce interventive births (15). The 2020 Chines Clinical Practice Guidelines for Normal Birth further encouraged the use of mobility and free positions to improve birth outcomes (16).

### 3.2 Classification of free positions

**Figure 1:**
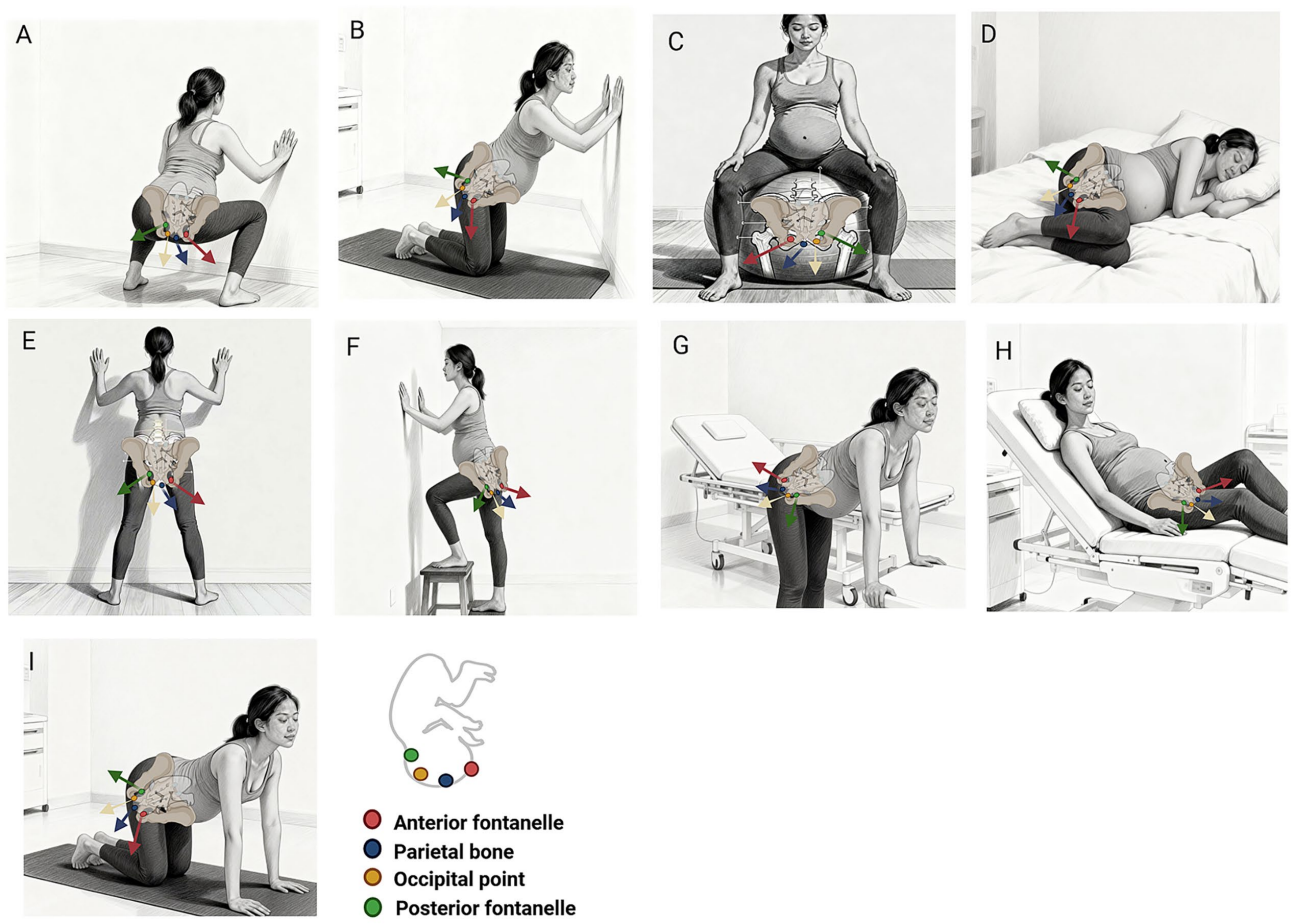
Schematic illustration of free positions during the second stage of labor. **(A)** Squatting position: this position induces pelvic anteversion, leading to posterior displacement of the sacrum and coccyx, and inferior movement of the pubic symphysis. This dynamically enlarges the pelvic outlet dimensions. The strong gravitational vector facilitates fetal descent, while the flatter, elongated state of the pelvic floor muscles may reduce soft tissue resistance and distribute expulsive forces more evenly, thereby minimizing the risk of focal overstretching and neuromuscular damage. **(B)** Kneeling position: by freeing the sacrum from contact with the bed, this position allows for sacral recoil, increasing the posterior space within the pelvis. This is particularly advantageous for correcting occipito-posterior (OP) fetal positions and alleviating associated back pain, potentially reducing the need for instrumental delivery and its associated pelvic floor trauma. **(C)** Sitting position (e.g., on a birth ball): provides a balance between gravity assistance and maternal rest. The mobility afforded by the ball enables subtle pelvic movements (rocking, swaying), which can encourage fetal head engagement and rotation into a more favorable position for descent, promoting a controlled, less traumatic delivery. **(D)** Lateral recumbent position. This position minimizes pressure on the sacrum and perineum, facilitating a more controlled and gradual descent of the fetal head. It is frequently associated with the lowest incidence of severe (third- and fourth-degree) perineal tears, as it distributes pressure evenly and may prevent excessive, rapid stretching of the perineal body. **(E)** Standing position: capitalizes fully on gravity to align the fetal axis with the birth canal, promoting efficient descent. It also helps prevent compression of the inferior vena cava, optimizing uteroplacental blood flow, which is crucial for fetal well-being during the expulsive efforts. **(F)** Lunge position: as an asymmetric stance, it creates a functional increase in the pelvic diameter on the side of the forward leg. This can be used therapeutically to create space for a fetal head that is asynclitic or malpositioned, aiding in its rotation and descent. **(G)** Forward-leaning position (standing or sitting): effectively shifts the center of gravity anteriorly, pulling the weight of the uterus away from the maternal spine. This significantly reduces pressure on the lumbosacral nerves and muscles, offering pronounced relief from back labor pain and potentially improving the efficiency of uterine contractions. **(H)** Semi-sitting/recumbent position: an intermediate option that offers better gravitational advantage than full supine positioning. It supports maternal comfort and allows for effective use of abdominal muscles during pushing, while still providing reasonable access for clinical assistance if needed. **(I)** Hands-and-knees position. This position fully reverses the gravitational pull on the fetus, which can aid in the rotation of an OP fetus to an occipito-anterior position. It maximally unloads the sacrum and is highly effective for relieving severe back pain. The even distribution of the fetal head’s pressure across a wider area of the pelvic floor may protect against focal overstretching and levator ani avulsion.

Free positions refers to the ability of laboring woman to choose comfortable position during the birthing process (18). These positions are diverse and include squatting, kneeling, lateral, standing, and semi-sitting positions, each offering distinct characteristics and benefits. In the squatting position (Figure 1A), the maternal pelvic outlet effectively expands, providing increased space for fetal descent. This position significantly enhances the angle of advancement for the fetal head through the pelvic axis, cervix, and pelvic floor soft tissues, thereby reducing pressure and potential injury to the pelvic floor tissues (12). Squatting not only enlarges the pelvic outlet (as visualized by the posterior sacral movement in Figure 1A) and enhances pelvic mobility but also strengthens the abdominal, back extensor, and lower limb muscles (19). By leveraging gravity, fetal descent is facilitated, potentially shortening the duration of labor (20). In the kneeling position (Figure 1B), the dimensions of the mid-plane and pelvic outlet increase by approximately 1 cm (13). A biomechanical study utilizing 3D computational reconstruction from ultrasound images demonstrated that when the mother transitions from a supine to a legs-flexed position, the rotation angle of the pubic symphysis dynamically adjusts, resulting in an increase in width by approximately 1 mm (21). Research on the sitting position (Figure 1C) demonstrates that for parturients without epidural analgesia, the use of a birth ball facilitates flexible adjustment of sacral position, promotes fetal head rotation and descent, and significantly reduces visual analog scale (VAS) pain scores by 1.7 points (22) while shortening the second stage of labor by 21.12 min (11). Furthermore, birth ball application is associated with an 11% increase in the probability of vaginal delivery, without compromising delivery modes or increasing obstetric complication risks (23), establishing this method as a safe and effective non-pharmacological adjunct for intrapartum management. The lateral position (Figure 1D) is characterized by relative comfort and has been shown to mitigate maternal fatigue (24). This position alleviates pressure on the sacrum (25), promotes pelvic flexibility, and facilitates outlet expansion (11). The standing position (Figures 1E–G) capitalizes on gravitational forces, thereby accelerating fetal descent (26). Additionally, this posture helps prevent compression of intra-abdominal vessels, particularly the inferior vena cava (27). The semi-sitting position (Figure 1H) effectively harnesses gravity while maintaining a level of comfort for the mother. A study employing the VAS to assess pain levels indicated that discomfort in the semi-sitting position (mean score of 3.4) was significantly lower than in the supine position (mean score of 7.86) (*p* < 0.05) (28). In this position, the mother’s abdominal muscles are better engaged in the pushing process, which may contribute to a reduction in the duration of the second stage of labor, improved Apgar scores, and enhanced maternal comfort (29). The hands-and-knees position (Figure 1I) enables maternal mobility through actions such as rocking, swaying, and crawling, which promote an anterior shift of the fetal center of gravity, facilitate the widening of the pelvic outlet, and support fetal descent and internal rotation (11). Additionally, by alleviating lumbosacral pressure, this position enhances the available space for fetal movement within the uterus, thereby mitigating pain in the mother’s lumbosacral region during contractions and improving her overall comfort (30). Figure 1 schematically summarizes the studied free positions, with annotations highlighting their distinct anatomical alignments and proposed biomechanical actions relevant to pelvic floor protection.

## 4 Comparison between free positions and traditional positions: a layered perspective

It is critical to recognize that “free positions” constitute a heterogeneous category, and their effects may vary significantly depending on specific clinical circumstances, the particular position adopted, and maternal-fetal characteristics. This section synthesizes the evidence by considering these key modifiers, moving beyond a simplistic unified conclusion. Furthermore, it is important to acknowledge at the outset that the evidence base itself is characterized by significant heterogeneity in study designs (e.g., randomized controlled trials vs. observational studies), sample sizes, and definitions of critical outcomes (e.g., perineal trauma severity, criteria for pelvic floor dysfunction). This variability should be considered when interpreting the summarized findings.

### 4.1 Key modifying factors: parity, epidural analgesia, and specific positions

Crucial factors such as parity, epidural analgesia, and specific positions may significantly influence the effectiveness of free positions delivery. A recent prospective cohort study conducted among Chinese primiparous women demonstrated that sitting position during the second stage was associated with significantly shorter duration (50 min vs. 76 min, *p* < 0.001), higher rate of spontaneous vaginal birth (93.4% vs. 75.0%), lower episiotomy rate (23.1% vs. 46.7%), and more positive childbirth experience compared to the lithotomy position (31). Similarly, a cross-sectional study reported that primiparas using lateral positions had better childbirth experiences (e.g., higher sense of control and safety) and a lower proportion of second-degree perineal tears compared to those in lithotomy positions. However, among multiparous women, neither sitting nor lateral positions showed significant advantages over lithotomy positions in terms of labor duration, delivery mode, perineal outcomes, or childbirth experience (32). These findings suggest that the benefits of free positions delivery may be more pronounced in primiparous women. In addition, in nulliparous women without epidural analgesia, upright positions (a subset of free positions in which the mother is vertical, including squatting, standing, and kneeling) like squatting and standing have been more consistently associated with a shorter second stage of labor and reduced rates of instrumental delivery compared to supine positions (11, 33). However, for women with effective epidural analgesia, the advantage in labor duration may be attenuated due to diminished sensation and mobility. In these cases, alternative free positions such as the lateral position or supported sitting become particularly valuable, primarily for enhancing maternal comfort and potentially reducing the risk of severe perineal trauma by allowing more controlled, spontaneous pushing (24, 34). Moreover, different positions exert distinct biomechanical effects, suggesting they may be optimally employed for specific indications. For example, the hands-and-knees has shown promise in facilitating rotation of the fetal head from an OP position to a more favorable occipito-anterior position, potentially reducing back pain and the need for operative intervention (30, 33). Additionally, the lateral recumbent position is frequently associated with the lowest incidence of severe perineal tears (third- and fourth-degree) (35, 36), likely due to a more gradual and controlled descent of the fetal head that distributes pressure evenly across the perineum. Positions that increase flexion at the hips and lumbar spine, such as deep squatting, are theorized to maximally enlarge the pelvic outlet (13, 19), a benefit most evident when maintained during the active pushing phase.

### 4.2 Timing and duration of position changes

Evidence regarding the impact of maternal position on labor duration must be interpreted through the lens of the modifying factors described above. Research has demonstrated that adopting free positions during the second stage of labor can significantly shorten its duration (37). Specifically, when this stage lasts less than one hour, the risk of perineal tearing is reduced; however, the risk increases when the duration exceeds two hours. Importantly, after three hours, the risk of tearing does not further escalate (38). Furthermore, upright position can substantially enhance maternal agency during childbirth, mitigate labor pain, and contribute to a shorter second stage of labor (33, 39). This is attributed to the ability of these free positions to allow the mother to effectively utilize the body’s natural forces, thereby facilitating smoother fetal descent and expediting the birthing process. Nonetheless, some studies have indicated that while the general consensus supports the efficacy of free positions in reducing the duration of the first stage of labor, several of the 25 studies reviewed did not show statistically significant differences (40). This lack of significance is attributed to limited sample sizes and inadequate standardization of interventions. In specific studies, the difference in labor duration between the group employing free positions and the recumbent group was within ± 30 min, which did not constitute a clinically significant reduction. During the early stages of labor, uterine contractions have not yet reached their peak intensity, leading to a relatively gradual descent of the fetus. Consequently, changes in maternal position during this phase do not significantly facilitate the progression of labor, resulting in comparable durations between the two positions (41).

### 4.3 Rate of spontaneous vaginal delivery

Free positions during labor have been associated with higher rate of spontaneous vaginal delivery in some studies (42). However, this effect may be limited in women with structural abnormalities of the birth canal, where the atypical relationship between fetus and pelvis may necessitate cesarean sections or instrumental assistance regardless of position (43). Some studies have found no statistically significant difference in spontaneous delivery rates between upright and traditional positions when anatomical challenges are present (40).

### 4.4 Incidence of perineal tears and episiotomy

Adopting free positionsduring labor has been shown to significantly decrease the incidence of perineal tears and episiotomies in several studies (35, 36). However, this effect may be modified by factors such as fetal macrosomia,which constitutes an independent risk factor for severe perineal tears and not be fully mitigated by positional changes alone (44, 45). Consequently, in these circumstances, there is no statistically significant difference in the incidence of perineal tears and episiotomy between free and conventional positions. It is important to acknowledge that the evidence on perineal trauma arises from studies with heterogeneous designs, varying definitions of tear severity, and different episiotomy policies. This heterogeneity may contribute to inconsistent findings and should be considered when interpreting the overall protective effect of free positions.

### 4.5 Impact on pelvic floor function

#### 4.5.1 Degree of pelvic floor muscle stretching

Evidence regarding the impact of free positions on objective measures of pelvic floor function is evolving. Some studies have suggested an association between free positions and reduced incidence of pelvic organ prolapse (POP) (46, 47). Imaging evidence indicates that damage to the iliococcygeal muscle, which is crucial for pelvic floor support, is significantly underestimated during conventional supine examinations. Its true extent of injury can only be adequately assessed through upright MRI (48). A prospective ultrasound study involving 78 primiparous women found that during the active first stage of labor, the transverse diameter of the pelvic floor hiatus exhibited only a slight but significant increase (from 39.4 mm to 44.3 mm, *p* < 0.01), and this change was correlated with the degree of fetal descent (49). These imaging findings highlight the importance of the second stage of labor as a critical period for pelvic floor loading.

#### 4.5.2 Incidence of urinary and fecal incontinence

Free positions have been shown to significantly decrease the incidence of urinary and fecal incontinencein some studies. Specifically, free positions were associated with a significantly lower rate of urinary incontinence (40.5% compared to 48.9%, *p* = 0.03), particularly in cases of stress urinary incontinence (50). Research indicates that the incidence of stress urinary incontinence following delivery in a lateral position was only 11%, in contrast to 23% for those in a supine position (*p* < 0.05) (51). However, the available studies employ diverse methodologies, sample sizes, and follow-up durations, which limits direct comparability. Future research with standardized outcome measures and longer follow-up is needed to strengthen causal inferences.

### 4.6 Impact on fetal and neonatal outcomes

In the context of childbirth, adopting free positions during labor, characterized by frequent positional changes, can significantly influence the dynamic mechanics of the pelvis. Thisapproach facilitates continuous adaptation between the fetus and the pelvic structure, thereby enhancing the rotation of the fetal head and correcting any abnormal fetal presentations. Evidence suggests that free positions are advantageous, as they are associated with lower rates of episiotomy, despite a slight increase in the incidence of first- and second-degree perineal tears. Additionally, these positions correlate with reduced rates of instrumental vaginal deliveries and diminished signs of stress in neonates (33). Furthermore, the free positions enhance the mother’s perception of control during labor and are linked to improved Apgar scores for newborns. The lateral position, in particular, has been shown to mitigate the risk of umbilical cord compression and to enhance the acid–base balance in neonates (52). The combined effects of gravity and amniotic fluid facilitate the descent of the fetal head and cervical dilation, allowing for the fetal head to navigate along the physiological axis of the pelvis. This mechanism not only aids in correcting fetal position but also reduces the duration of labor, accelerates labor progression, enhances neonatal outcomes, and increases the likelihood of spontaneous vaginal delivery. The key comparative findings regarding labor duration, perineal and pelvic floor outcomes, and neonatal safety across the different positioning categories are synthesized in Table 1.

**Table 1:** Comparative summary of key maternal and neonatal outcomes associated with different maternal positions during the second stage of labor.

Position category/specific position	Duration of second stage	Perineal trauma	Pelvic floor outcomes (short-term)	Neonatal outcomes	Key considerations/modifying factors
Free positions (Collective)	↓ or ↔ (Trend towards shortening, but evidence is heterogeneous)	↓ Incidence of episiotomy and severe (3rd/4th degree) tears. ↔ or slight ↑ in 1st/2nd degree tears.	↓ Incidence of short-term postpartum urinary incontinence. Potential to reduce pelvic floor muscle overstretching.	↔ Apgar scores. ↓ Signs of fetal stress. ↓ Rate of instrumental delivery.	Benefits most pronounced in nulliparous women without epidural. Heterogeneity within the category is significant.
Squatting	↓ (Leverages gravity, strengthens expulsive muscles)	Potential for even pressure distribution, but may be less protective than lateral position for severe tears.	Associated with pelvic outlet expansion and optimized fetal descent angle.	Generally comparable to other spontaneous delivery positions.	Requires significant maternal strength and mobility. Often unsuitable with epidural analgesia.
Hands-and-Knees	↔ or ↓(Particularly for rotation of occiput posterior (OP) position)	Evidence specific to perineal protection is less consistent.	Primary benefit linked to facilitating fetal rotation, reducing obstructed labor.	Beneficial for correcting OP position, thereby reducing operative interventions.	First-line recommendation for persistent OP position and maternal back pain.
Lateral (Recumbent)	↔ or ↓ (Allows for controlled, spontaneous pushing)	↓↓ Strongest evidence for reducing severe (3rd/4th degree) perineal tears.	↓ Significant reduction in postpartum urinary incontinence.	↓ Risk of umbilical cord compression. Improved acid–base balance.	Ideal for women with epidural analgesia, fatigue, or hypertension. Promotes controlled descent.
Sitting/semi-sitting	↓ (Facilitated by gravity and engagement of abdominal muscles)	↔	Contributes to maternal comfort, which may indirectly support effective pushing.	↔	Use of a birth ball can enhance flexibility and comfort. Suitable for a wide range of women, including some with epidural.
Traditional positions (Lithotomy/Supine)	↔ or ↑ (May prolong stage due to non-physiological axis and reduced gravity assistance)	↑ Incidence of episiotomy. ↑ Risk of perineal tears due to focalized pressure.	↑ Risk of short-term urinary incontinence. Concentrated mechanical stress may increase neuromuscular injury.	↔ or slight ↑ in rate of instrumental delivery. ↑Risk of non-reassuring fetal heart rate patterns.	Common in clinical practice but may concentrate stress on the posterior perineum. Facilitates monitoring and intervention but is non-physiological.

Symbols: ↓ Decrease/lower risk/shorter duration (favorable outcome). ↑ Increase/higher risk/longer duration (unfavorable outcome). ↔ No significant difference or inconsistent findings reported. ↓↓/↑↑ Strong evidence for a marked decrease or increase.

## 5 Biomechanical and molecular mechanisms

### 5.1 Disadvantages of traditional positions

**Figure 2:**
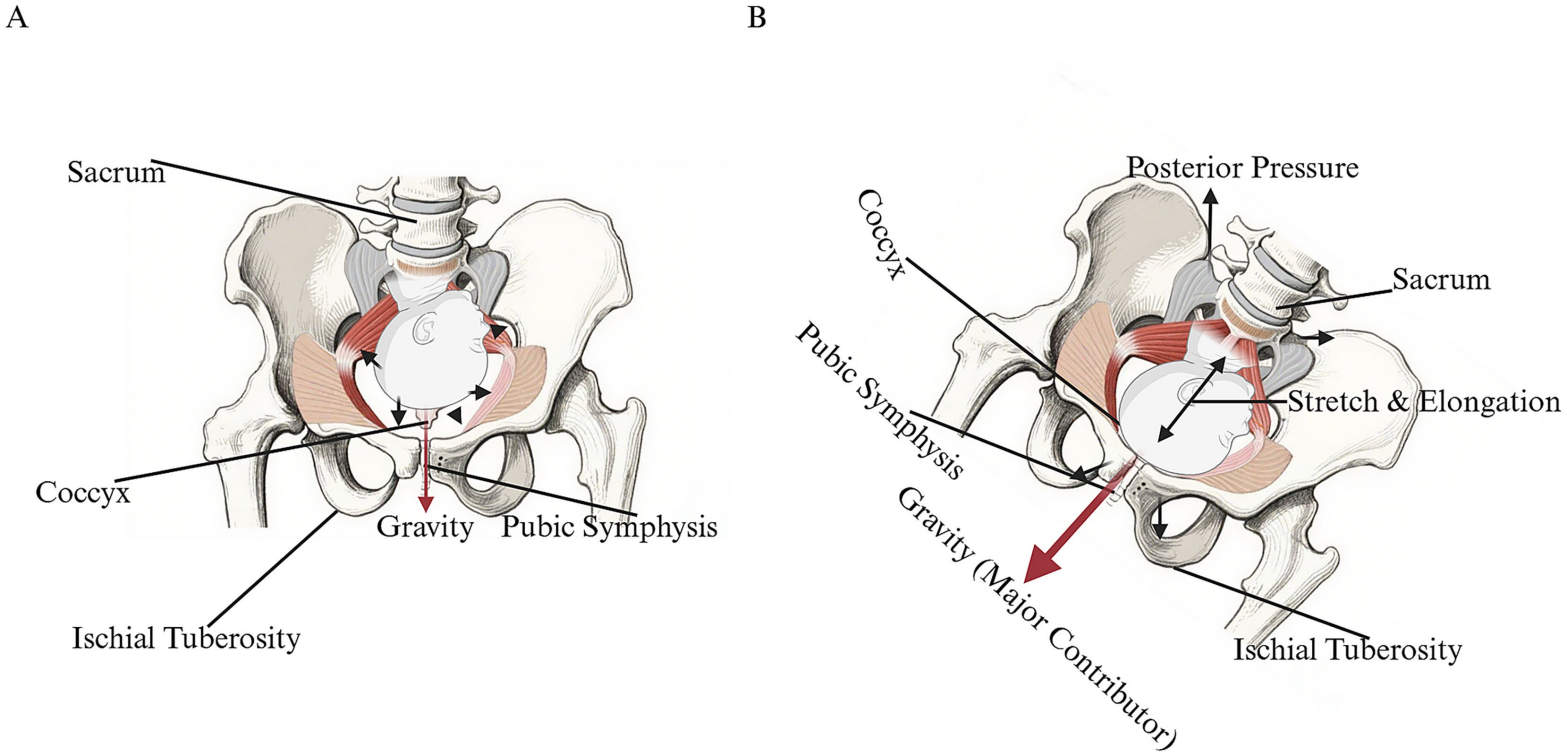
Comparative schematic of the pelvic skeleton in the supine and squatting positions. **(A)** Supine position (lithotomy position). In the supine position, the pelvis maintains a neutral anatomical alignment. The sacrum remains relatively fixed against the birthing surface, limiting posterior mobility, while the pubic symphysis is positioned superiorly, constraining the anteroposterior diameter of the pelvic outlet. Gravitational assistance (short red arrows) for fetal descent is minimal. The pelvic floor muscles assume a relaxed, “hammock-like” configuration, providing passive support but offering increased resistance to fetal passage. The fetal head remains relatively higher within the birth canal due to the suboptimal mechanical axis. **(B)** Squatting position. The squatting position induces significant dynamic changes: pelvic anteversion causes posterior displacement of the sacrum and coccyx, and the pubic symphysis moves inferiorly, collectively expanding the pelvic outlet dimensions. The ischial tuberosity acts as a stabilizing fulcrum. Gravity (long red arrows) acts as a primary driving force, synergizing with uterine contractions to facilitate fetal descent. Pressure from the advancing fetal head (black arrows) further promotes sacrococcygeal movement. The pelvic floor muscles are stretched into a flatter, elongated state, reducing soft tissue resistance. These biomechanical adaptations align the fetal head closer to the outlet, simulating crowning and optimizing the efficiency of birth.

Conventional lithotomy and similar supine positions concentrate load on discrete pelvic floor regions, drive tissue stretch beyond physiological limits, and impair perfusion, thereby increasing the risk of microtrauma and ischemia. In the lithotomy position, extreme abduction and elevation of the legs focus pressure on specific pelvic floor areas, causing muscle stretch to exceed normal ranges; prolonged maintenance of this posture makes muscle fibers susceptible to tearing and may contribute to PFD, including pelvic organ prolapse, fecal incontinence, and urinary incontinence (36). Excessive stretching and compression can also hinder pelvic floor blood flow, reducing nutrient delivery and compromising muscle function (8). The biomechanical constraints of the supine position are illustrated in Figure 2A, which contrasts with the advantageous squatting position (Figure 2B).

### 5.2 Advantages of free positions

**Figure 3:**
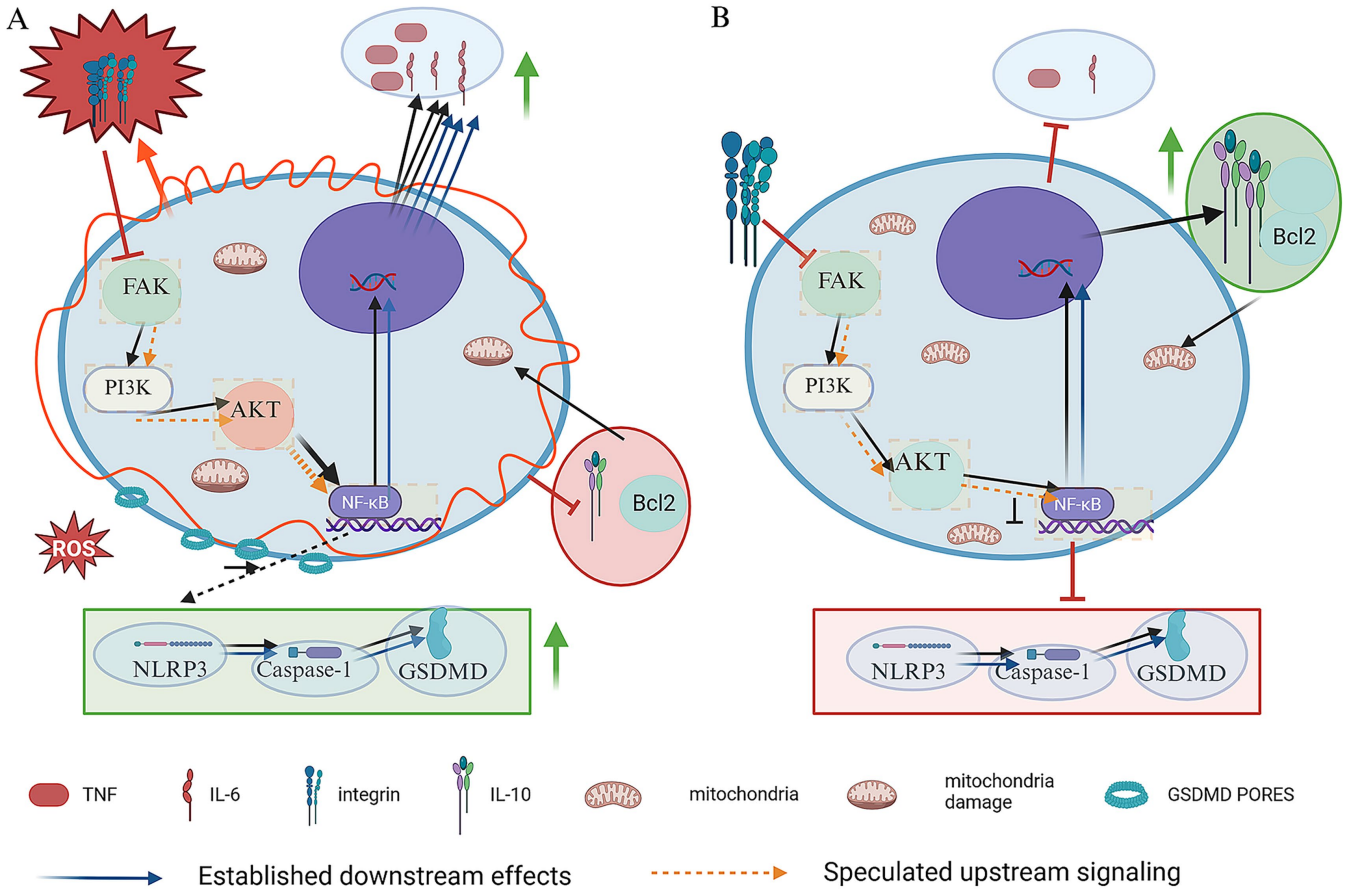
Hypothesized comparative biomechanical and molecular mechanisms of lithotomy position and free position delivery. **(A)** Supine position (lithotomy position). The diagram illustrates a hypothesized cascade of molecular events potentially induced by excessive mechanical stress (red jagged arrows). This stress is proposed to induce aberrant overactivation of the integrin–focal adhesion kinase (FAK) signaling pathway, which may subsequently trigger the PI3K/Akt pathway, and may lead to the degradation of IκB, promoting the nuclear translocation of nuclear factor kappa B (NF-κB). The connection from mechanical stress through Integrin/FAK to PI3K/Akt/NF-κB activation is depicted with a dashed arrow pathway and highlighted in orange, representing a hypothetical link based on mechanobiology studies in non-placental cell types. Within the nucleus, NF-κB drives the overexpression of pro-inflammatory cytokines (TNF-*α*, IL-6; green upward arrows) and activates the NOD-like receptor protein 3 (NLRP3)/Caspase-1/gasdermin D (GSDMD) pyroptosis pathway. The downstream consequences of NF-κB activation, including cytokine production and the execution phase of pyroptosis (NLRP3/GSDMD), are depicted with solid arrows and highlighted in blue, representing more directly supported pathological events in inflammatory contexts. This ultimately results in the formation of GSDMD pores on the cell membrane and triggering inflammatory cell death. Meanwhile, protective mechanisms are suppressed, with downregulated expression of the anti-inflammatory cytokine (IL-10) and anti-apoptotic protein (Bcl-2; red downward arrows and “┴” inhibition symbols), and cellular energy metabolism is impaired. **(B)** Free position delivery. The diagram illustrates the potential physiological signaling and protective outcomes mediated by appropriate mechanical stress. Physiological activation of the integrin–FAK (depicted as part of the same hypothetical upstream pathway, with dashed arrows/orange highlight) and PI3K/Akt signaling pathway is shown to inhibits IκB degradation, thereby effectively preventing the nuclear translocation of NF-κB. This inhibitory effect downregulates the expression of pro-inflammatory cytokines (TNF-α, IL-6) and suppresses the pyroptosis pathway (red “┴” inhibition symbols). Meanwhile, the expression of the anti-inflammatory cytokine (IL-10) and anti-apoptotic protein (Bcl-2) is upregulated (green upward arrows), and cellular energy metabolism is improved.

Upright and maternal-choice (“free”) positions redistribute forces and harness gravity to facilitate fetal descent, reducing pelvic floor loading. From a biomechanical perspective, these adjustments are well-documented (13). Squatting leverages gravity to aid fetal descent and distributes pelvic floor pressure more evenly, decreasing the muscular effort required for expulsion (13). In standing positions, gravity similarly promotes movement toward the pelvic outlet, reducing the load borne by pelvic floor muscles. The lateral position minimizes pressure on the sacrum and perineum, facilitating a more controlled and gradual descent of the fetal head (35, 36). The hands-and-knees position enables anterior shift of the fetal center of gravity, promotes widening of the pelvic outlet, and may aid in rotation from occipito-posterior to occipito-anterior position (11, 30). Clinically, allowing mothers to adjust posture based on somatic feedback can optimize pelvic diameters to accommodate the fetal head, reducing uneven pressure and excessive strain on pelvic floor muscles and promoting postpartum functional recovery (53). These biomechanical adaptations—enlargement of pelvic outlet dimensions, optimization of fetal descent angle, and more even distribution of expulsive forces—represent the best-established mechanisms by which free positions may confer protective effects on pelvic floor structures. Figure 3B illustrates these advantageous biomechanical features, in contrast to the lithotomy position shown in Figure 3A.

Beyond these biomechanical effects, emerging evidence from non-obstetric fields has raised the question of whether mechanical stimuli might influence tissue inflammation through cellular signaling pathways. Figure 3 presents a conceptual framework integrating these biomechanical observations with hypothetical molecular cascades. The molecular pathways depicted—including integrin–focal adhesion kinase (FAK), PI3K/Akt, and nuclear factor kappa B (NF-κB) signaling—are extrapolated from studies in other tissues (e.g., skeletal muscle, cardiovascular tissue, chondrocytes) and should not be interpreted as established mechanisms in childbirth. A key study in human skeletal muscle demonstrated that massage—a form of mechanical manipulation—activates the mechanotransduction pathway integrin–focal adhesion kinase (FAK) and attenuates nuclear factor kappa B (NF-κB) activation, thereby reducing pro-inflammatory cytokine production (54). Parallel evidence from exercise physiology and osteoarthritis models has shown that favorable mechanical stimuli can attenuate inflammation, oxidative stress, and cell death through shared signaling hubs such as NF-κB (55–57). In osteoarthritis models, exercise-induced Metrnl release inhibits PI3K/Akt/NF-κB signaling and the NLRP3/caspase-1/GSDMD pyroptosis pathway, protecting chondrocytes (58). Given that NF-κB is a known regulator of labor-associated inflammation (58), it is plausible, though entirely speculative, that the mechanical forces generated during free positions delivery could similarly modulate inflammatory responses in pelvic floor tissues. It is critical to emphasize that this FAK-NF-κB hypothesis remains a conceptual framework only. As illustrated hypothetically in Figure 3A (lithotomy) versus Figure 3B (free positions), excessive mechanical stress is speculated to aberrantly activate pro-inflammatory cascades (NF-κB, NLRP3 pyroptosis), while physiologically appropriate mechanical stimuli may theoretically inhibit these pathways and upregulate protective factors. However, direct evidence linking maternal position during childbirth to these specific molecular cascades in human pelvic floor tissues is currently lacking. The pathways shown in Figure 3 are presented as a conceptual framework—grounded in mechanistic studies from other fields—to generate hypotheses for future translational research, including biomarker analyses during labor in different positions and appropriately designed animal models (55–58).

### 5.3 Mechanisms of pelvic floor dysfunction occurrence

The pathogenesis of PFD is complex, with pregnancy and childbirth being key triggers. During pregnancy, increased uterine pressure and hormonal changes relax pelvic floor support structures. Vaginal delivery can injure pelvic floor muscles, fascia, and nerves (7). Studies in pregnant mice show significant changes in pelvic floor tissue structure (59), while. Postpartum guinea pig studies indicate oxidative stress damage (60, 61). Additionally, chronic factors like coughing, constipation, heavy physical labor, and obesity also weaken pelvic floor tissues by increasing abdominal pressure. Metabolic abnormalities in the extracellular matrix (ECM) lead to reduced collagen synthesis and increased degradation by matrix metalloproteinase (MMP), weakening pelvic floor support structures (60). Furthermore, oxidative stress generates excessive reactive oxygen species (ROS) that activate inflammatory pathways releasing interleukins and tumor necrosis factor, which can damage pelvic floor tissues (62).

## 6 Maternal satisfaction and comfort

### 6.1 Maternal satisfaction surveys

Maternal satisfaction serves as a crucial indicator in the management of labor. Research indicates that mothers who adopt free positions during delivery report significantly higher levels of satisfaction with the birthing process compared to those who utilize traditional positions (51, 61). Furthermore, a qualitative study conducted in Germany revealed that mothers who autonomously selected their birth positions experienced markedly greater satisfaction with the delivery compared to those who did not exercise such autonomy (63).

### 6.2 Reasons for improved comfort

Free positions have been associated with reduced maternal pain and enhanced comfort during labor. Allowing women to autonomously choose their positions enhances relaxation, facilitates identification of comfortable pushing techniques, and enables posture modification in response to physical and emotional cues (11, 64). This flexibility helps prevent fatigue and reduce psychological stress associated with prolonged maintenance of a single position.

## 7 Application of free positions in clinical practice

### 7.1 Challenges faced

Despite the documented advantages of free positions in research, their implementation in clinical practice continues to encounter significant challenges. Some midwives and obstetricians’ express concerns regarding the safety and efficacy of these positions, and may also lack appropriate training (65, 66). A study conducted by Musie et al. (67) revealed that both undergraduate and postgraduate midwifery programs inadequately address the necessary skills and training, leading to midwives’ insufficient competence in employing alternative birth positions in clinical settings.

### 7.2 The impact of education on maternal choice

Promote widespread adoption of free positions requires multifaceted strategies. First, specialized training programs for healthcare professionals are essential to improve understanding of benefits, techniques, and safety considerations (68). Second, maternal educating through midwifery care models incorporating postural and psychological interventions has been shown to reduce anxiety, increase spontaneous delivery rates, and improve outcomes (69, 70). Third, institutional support through facilities upgrades—including adjustable beds and adequate space—is necessary to enable mobility and position changes during labor (71). Implementing such measures is crucial for addressing the challenges associated with free positions in clinical practice.

### 7.3 Content and methods of education

Healthcare providers should offer detailed information about free positions during pregnancy, including potential advantages, implementation techniques, and strategies for autonomous position selection. Prenatal classes, educational materials, and visual aids (images, videos) can enhance maternal understanding (72, 73).

Importantly, the foundational role of structured antenatal education in promoting positive birth behaviors is well demonstrated. For instance, a randomized controlled trial conducted in Jordan showed that a childbirth preparation course significantly increased rates of spontaneous labor onset, improved cervical dilation at admission, and facilitated earlier initiation of breastfeeding among nulliparous women (74). Although this study did not specifically evaluate maternal positioning during labor—all participants delivered in the lithotomy position as per local protocol—its findings underscore how antenatal education empowers women through knowledge acquisition, confidence building, and the development of practical coping skills. By integrating targeted instruction on free positions into such educational frameworks, healthcare providers can further encourage informed choice and uptake of these evidence-based practices during labor.

Research indicates that most midwives consider free positions appropriate for women meeting specific criteria: singleton fetus in cephalic presentation, full-term gestation (37–42 weeks), normal fetal heart rate, adequate maternal endurance, and suitability for vaginal delivery (75, 76). Implementation should align with labor dynamics and may be particularly beneficial in cases of prolonged fetal descent or inadequate uterine contractions.

### 7.4 Safety considerations and evidence-based contraindications

The safe implementation of free positions during labor requires distinguishing between evidence-based contraindications and perceived clinical challenges. Evidence-based contraindications are grounded in the need for continuous fetal monitoring or readiness for urgent intervention, as outlined in international guidelines. For instance, non-reassuring fetal status necessitates continuous electronic monitoring, best facilitated in lateral or supine positions according to WHO recommendations (15). Similarly, severe maternal hemodynamic instability (e.g., active hemorrhage or severe preeclampsia) or scenarios requiring expedited delivery are recognized as contraindications (14). Conversely, many perceived barriers to free positions delivery are rooted in practice culture, training gaps, or logistical constraints rather than safety evidence. Surveys consistently report concerns related to maternal exhaustion, epidural analgesia management, and lack of practitioner confidence as primary obstacles (65, 66, 75). These should be reframed as modifiable challenges. For instance, while epidural analgesia alters mobility, it does not preclude safe use of supported positions like lateral or sitting, which have demonstrated benefits in randomized trials (34). Common conditions such as well-controlled gestational diabetes or chronic hypertension, in the absence of acute complications, do not constitute evidence-based reasons to restrict maternal position choice (15). Practitioner inexperience and inadequate physical environments (e.g., non-adjustable beds, limited space) are system-level barriers that must be addressed through education and facility design, not misapplied as patient safety limitations (67). Differentiating between true safety concerns and logistical challenges is essential to avoid inappropriately limiting maternal choice.

## 8 Discussion

A critical synthesis of the reviewed literature reveals a distinct gradient in the certainty of evidence across different outcome domains and time horizons, an assessment guided by the principles of the GRADE framework as described in the Methods. For short-term obstetric outcomes—specifically, the duration of the second stage of labor, rates of spontaneous vaginal delivery, and the incidence of severe perineal trauma—the body of evidence can be considered of moderate to high certainty. This conclusion is supported by a number of randomized controlled trials and meta-analyses, which, despite some heterogeneity, provide a consistent direction of effect (11, 33, 36). However, it is important to note that even among these studies, definitions of outcomes such as “severe perineal trauma” vary, with some using third-degree tears alone and others including fourth-degree tears or episiotomy as composite outcomes. This variability in outcome definitions may contribute to the heterogeneity observed and should be considered when interpreting pooled estimates. In contrast, the evidence concerning long-term pelvic floor endpoints—such as the development of urinary incontinence, fecal incontinence, or pelvic organ prolapse one year or more postpartum—is currently of low certainty. The available data are predominantly observational, with methodological limitations, varying definitions (49, 50), and most importantly, inadequate duration of follow-up to assess sustained dysfunction (34, 51). This lack of standardization in outcome measurement across studies severely limits the ability to synthesize evidence or draw firm conclusions about long-term protective effects. While the biomechanical rationale for a protective effect of free positions is compelling (12, 13), the clinical evidence for sustained, long-term benefit is not yet fully established and must be considered preliminary. This graded certainty—moderate/high for short-term procedural benefits versus low for long-term protective effects—must inform the interpretation of our findings and any resulting clinical recommendations. A summary of this evidence grading for key outcomes is presented in Table 2.

**Table 2:** Certainty of evidence for key outcomes associated with free positions delivery.

Outcome domain	Specific outcome	Direction of association	Certainty of evidence (GRADE-informed)	Basis for grading (representative references)
Short-term obstetric outcomes	Duration of second stage	Reduction (↓)	Moderate	Meta-analyses of RCTs show a trend, with some inconsistency (11, 33)
	Spontaneous vaginal delivery	Increase (↑)	Moderate-High	Consistent findings from RCTs and systematic reviews (33, 42).
	Severe (3rd/4th degree) perineal tears	Reduction (↓)	Moderate	Supported by cohort studies and RCTs; effect is position-specific (36, 46).
Pelvic floor outcomes (mid-term)	Postpartum urinary incontinence (≤1 year)	Possible reduction (↓)	Low-moderate	Primarily observational studies with inconsistent results (49, 50).
Pelvic Floor outcomes (long-term)	Persistent urinary incontinence / pelvic organ prolapse (>1 year)	Unclear / insufficient evidence (↔)	Low	Lack of dedicated long-term studies; evidence is indirect and insufficient (34, 51).
Maternal experience outcomes	Birth satisfaction and autonomy	Increase (↑)	High	Consistent evidence from qualitative and quantitative studies (51, 62, 63).
Neonatal safety outcomes	Apgar scores	No significant difference (↔)	High	Consistently reported across studies, indicating safety (30, 33).

Certainty of evidence was assessed narratively by the authors, informed by GRADE principles (17). High: consistent findings from high-quality randomized controlled trials (RCTs). Moderate: evidence from RCTs with limitations, or strong, consistent observational evidence. Low: evidence from observational studies with serious limitations, indirect evidence, or is very inconsistent (encompassing the GRADE ‘low’ and ‘very low’ categories). Symbols: ↑ Favorable increase; ↓ Favorable decrease; ↔ No clear association/insufficient evidence.

Intrapartum ultrasound technology enables real-time assessment of fetal position, cervical dilation, and conditions of the birth canal. When integrated with adjustments to the maternal free positions, this technology has the potential to enhance the labor process. A study (77) investigated the differences in intrapartum ultrasound measurements between mothers positioned laterally and those in semi-recumbent positions, revealing only minor variations in the angle of progression (AOP) and head-perineum distance (HPD). These findings suggest that monitoring with intrapartum ultrasound is feasible in free positions. However, it is important to recognize that the adoption of both free positions and these novel technologies is heavily influenced by institutional culture and resource availability. In settings where lithotomy positions remain the institutional standard—often due to bed design, monitoring protocols, or clinician training—the feasibility of implementing both free positions and adjunctive technologies may be limited. This contextual variability means that study findings from well-resourced, midwifery-led settings may not directly translate to other clinical environments. Additionally, virtual reality (VR) technology provides immersive experiences that may assist mothers in alleviating pain and anxiety during labor. A randomized controlled trial demonstrated (78) that mothers utilizing VR devices reported significantly lower pain scores during early labor (from 2.6 ± 1.2 to 2.0 ± 1.3), with 95% of participants indicating a willingness to use the technology again. Notably, this study was conducted in a single tertiary center, and the participants were predominantly nulliparous women with low-risk pregnancies, raising questions about the generalizability of findings to broader obstetric populations. Moreover, VR simulations of delivery scenarios, such as three-dimensional virtual delivery simulators, can aid healthcare providers in predicting changes in fetal position and optimizing delivery strategies. Another study of 105 first-time mothers found that lying down was the most common position in the 24 h before delivery, accounting for over 80% of the time (average 19.2 h). More than 90% delivered in non-sacral flexion positions (lying/sitting). Epidural analgesia users were significantly more likely to maintain these positions (42.6% vs. 21.3%, *p* = 0.03) (79). This finding highlights an important interaction between intrapartum interventions and positioning choices, suggesting that women with epidural analgesia may face additional barriers to adopting upright positions—a factor that warrants further investigation and targeted clinical strategies.

This narrative review has several limitations that should be considered when interpreting its findings. Firstly, as a narrative synthesis rather than a systematic review or meta-analysis, the selection and inclusion of evidence may be subject to author bias, and the conclusions lack the quantitative rigor of a pooled statistical analysis. Secondly, the majority of the cited clinical studies are observational or small-scale randomized controlled trials, which may be influenced by confounding variables and thus limit the strength of causal inferences. Third, the evidence synthesized comes from studies with considerable heterogeneity in design, population, outcome definitions, and clinical settings. This variability must be considered when interpreting the consistency and generalizability of findings, especially regarding perineal trauma and pelvic floor morbidity. Furthermore, the proposed molecular mechanisms, particularly those involving the FAK-NF-κB axis, are primarily extrapolated from experimental models in other organ systems; direct human evidence linking free positions to intrapartum inflammatory modulation remains scarce and warrants validation through targeted biomarker studies. Finally, the long-term benefits of free positions on pelvic floor function beyond the immediate postpartum period are not yet fully established, highlighting the need for more longitudinal research with extended follow-up.

## 9 Conclusion

The second stage of labor represents a pivotal period for pelvic floor health, with the selection of delivery position may influence subsequent pelvic floor function. Current evidence suggests that the adoption of free positions during this stage is associated with potential short-term benefits, including a trend toward reduced risk of perineal tears and lower rates of early postpartum urinary incontinence. These findings indicate that free positions may be considered as a valuable option within individualized labor management, particularly for women seeking to enhance their childbirth experience and potentially reduce short-term perineal morbidity. However, it is important to emphasize that the evidence for long-term pelvic floor protection remains limited and of low certainty. The heterogeneity of existing studies and the lack of standardized long-term outcome data mean that definitive conclusions cannot yet be drawn regarding the sustained benefits of free positions for pelvic floor health. Future research would benefit from long-term follow-up studies with extended follow-up periods (5 years, 10 years, or beyond) and standardized outcome measures. Such investigations could help determine whether the promising short-term benefits of free positions translate into enduring protection for pelvic floor function.
